# When combining injunctive and descriptive norms strengthens the hypocrisy effect: A test in the field of discrimination

**DOI:** 10.3389/fpsyg.2022.989599

**Published:** 2022-11-29

**Authors:** Maxime Mauduy, Daniel Priolo, Nicolas Margas, Cécile Sénémeaud

**Affiliations:** ^1^Université de Caen-Normandie, Laboratoire de Psychologie Caen Normandie (EA 7452), Caen, France; ^2^Université de Lausanne, Institut des Sciences du Sport, Lausanne, Switzerland; ^3^Université Paul Valéry Montpellier 3, Laboratoire EPSYLON (EA 4556), Montpellier, France

**Keywords:** induced-hypocrisy paradigm, injunctive norm, descriptive norm, discrimination prevention, Cyberball game

## Abstract

The induced-hypocrisy is a paradigm in which people promote a normative behavior (normative salience step) and then recall their past transgressions (transgression salience step). It is an effective two-step procedure for encouraging prosocial behaviors. This study aims to explore whether discrimination can be reduced using the hypocrisy paradigm combining two kinds of social norms, namely injunctive and descriptive norms. We assigned 80 participants to descriptive norm-related hypocrisy, injunctive norm-related hypocrisy, combined-norm hypocrisy, and control conditions. Results showed that intention to adopt active normative behaviors was higher in the combined-norms than in the single norm hypocrisy conditions. We observed the same pattern in reducing discriminatory behaviors in the Cyberball game, which measures passive discrimination (exclusion). Our findings have both practical and theoretical implications. First, they provide a new and effective means for producing behavioral changes in the field of discrimination. Second, they contribute to further investigating the explanatory processes underlying the hypocrisy effect.

## Introduction

Discrimination, defined as “the differential treatment of people on the basis of their membership in a given group” (Bodenhausen and Richeson, 2010, p. 343), is still a major societal issue in the 21st century. While many social groups are affected (e.g., elderly, homosexual, obese), immigrants and other people from foreign countries are commonly victims of discrimination (Kaas and Manger, 2012; Sasaki et al., 2017). In France, the occurrence of discrimination against foreign people is highlighted both in field surveys (e.g., DARES, 2021) and laboratory research (e.g., Mange et al., 2016; Gereke et al., 2020; Valfort, 2020). These discriminations have changed over the past few decades (e.g., Dovidio and Gaertner, 1996; Dovidio et al., 2002) from blatant and active forms (e.g., physical assaults, insults, Molero et al., 2013) to more subtle and passive forms (e.g., avoiding contact with the person, Cuddy et al., 2007; reducing the amount of time spent interacting with them, Hebl et al., 2002). This shift from active to passive discriminations would itself be explained by the shift in societies from a prodiscrimination social norm to an antidiscrimination social norm whereby it is socially unacceptable to discriminate against others (Kite and Whitley, 2016). According to Cialdini and Trost (1998), “social norms are rules and standards that are understood by members of a group, and that guide and/or constrain social behavior without the force of laws” (p. 152). The purpose of this study is to further investigate the influence of the anti-discriminatory social norm in preventing discrimination against foreign people.

### Discrimination prevention: Activating social norms or focusing people on deviance

A great deal of research has been done to develop and test discrimination prevention strategies (see Kite and Whitley, 2013 and Paluck and Green, 2009 for reviews, and Paluck et al., 2021 for a meta-analysis). Because the expression of prejudice and discriminatory behaviors depends on contextual salient social norms (e.g., Watson, 1950; Reitzes, 1953; Crandall et al., 2002; see Sechrist and Stangor, 2005 for a review), normative strategies are among the most effective for preventing discrimination (Paluck and Green, 2009; Paluck et al., 2021). A brief review of the literature leads us to classify normative strategies for preventing discrimination into two types. The first consists in activating the antidiscrimination social norm whereas the second consists of focusing people on their deviance from this norm. More precisely, the activation of social norms has been widely investigated by researchers as a first route in an attempt to reduce discrimination. For example, Monteith et al. (1996) showed that the expression of prejudice toward the black (Study 1) and gay population (Study 2) was reduced when participants were exposed to antidiscrimination beliefs of confederates versus pro-discrimination beliefs. Similarly, in a set of studies conducted by Falomir-Pichastor and his colleagues in Switzerland (Falomir-Pichastor et al., 2004, 2013; Gabarrot et al., 2009, Study 1) and France (Falomir-Pichastor et al., 2007; Gabarrot et al., 2009, Study 2) antidiscrimination social norm salience, through providing to participants results of so-called studies, influences discriminatory behaviors (i.e., distribution of funds towards French and immigrants; Falomir-Pichastor et al., 2004, Studies 2 and 3).

In parallel, a second range of research has set out to study not the exposure to the norm but the consequences of deviance from this norm (see Monteith et al., 2016 for a review). To this end, Devine et al. (1991) developed a particular tool: the “Should-Would Discrepancy questionnaire.” Following two steps, people are asked (1) how they think they should (according to members of their in group) behave in interactions with members of a discriminated out-group and then (2) how they think they would actually behave in these interactions. The results of several studies show that perceived discrepancy between what people should do and what they would really do in discrimination contexts arouses negative emotions (e.g., guilt, threat; Plant and Devine, 1998; Monteith et al., 2002; Fehr and Sassenberg, 2010). However, few studies show the practical relevance of this “Should-Would Discrepancy questionnaire” on effective reduction of discriminatory behavior (Amodio et al., 2007). Yet, people’s awareness of the gap between what is expected (the norm) and what is done (a behavior) is the cornerstone of a well-known paradigm – the Induced-Hypocrisy Paradigm (IHP, Aronson et al., 1991) – developed in the field of cognitive dissonance theory as applied to behavioral change (Festinger, 1957). It is this paradigm that we propose to use in this article as a tool for reducing discrimination.

### Reinforcing the deviance from the antidiscrimination norm to reinforce the hypocrisy effect

Induced hypocrisy (Aronson et al., 1991) is an efficient cognitive dissonance paradigm for encouraging normative behaviors in many fields (e.g., pro-environmental behaviors such as recycling waste, health behaviors such as use of condoms, see Liégeois et al., 2017 for a review, and Priolo et al., 2019 for a meta-analysis). In this two-step procedure, people promote a social norm (i.e., the “normative-salience step”) and then recall their own past failures to comply with it (the “transgressions-salience step”). Making salient this inconsistency generates the hypocrisy effect (Stone and Fernandez, 2008), leading people to adopt behaviors in accordance with the norm. When the IHP was created, the main and consensual explanation of its effect was self-consistency theory (Aronson, 1999), which focuses on the role of self-threat. However, more recent explanations, such as the deviation-from-norm approach (Liégeois et al., 2017), give social norms a central role in producing the hypocrisy effect.

This deviation-from-norm approach considers the behavioral change in the IHP as the reduction of the perceived gap between social normative beliefs and behaviors, which echoes previously cited writings by Devine. To give further proof of this, Priolo et al. (2016) showed that the hypocrisy effect could be obtained both when the first step made salient an injunctive norm (i.e., what most people approve of, Cialdini and Goldstein, 2004) or another type of social norms, i.e., the descriptive norm (i.e., what most people do). The results are consistent with their hypothesis and show that behavioral inconsistencies with descriptive or injunctive norms lead people to change their behavior (i.e., more donations for an ecological association). Besides, Liégeois et al.’s (2017) approach also assumes that the salience of social-normative beliefs is a key factor. The more people have access to their social normative beliefs, the higher the perception of discrepancy between normative beliefs and transgressions, and the greater the hypocrisy effect. Therefore, a way to enhance the hypocrisy effect would be to strengthen the role of social norms in the normative-salience step of IHP.

Although the IHP appears to be highly suitable for the prevention of discrimination, it has only been tested once in this field (Son Hing et al., 2002). Furthermore, following Liégeois et al.’s (2017) approach of the IHP, the effectiveness of the hypocrisy procedure should be increased upon strengthening its normative-salience step. As social norms’ theories, such as the theory of normative social behavior (Lapinski and Rimal, 2005; Chung and Rimal, 2016), and research (e.g., Kallgren et al., 2000) predict and show that the combination of injunctive norms and descriptive norms enhances the behavioral effect, we expect the hypocrisy effect to be enhanced by the combined activation of both injunctive and descriptive anti-discrimination norms. Indeed, perceiving our past behaviors as deviating not only from what the majority of people approve of but also from what they do (descriptive norm) should increase the deviation from normative beliefs and enhance behavior change. To test this hypothesis of an additive effect of combining two norms rather than one in the hypocrisy procedure, we conducted an experiment comparing a control group to three hypocrisy conditions (Descriptive norm-related hypocrisy vs. Injunctive norm-related hypocrisy vs. Combined-norm hypocrisy) on normative behavioral intention and on passive behaviors of discrimination.

## Materials and methods

### Sample

We collected data from 89 students who were not paid for their participation. Three participants were removed for errors in recording a measure, five for not recalling any transgression, and one for not recalling any transgression and suspicions related to a measure. Attrition was balanced across conditions. A total of 80 white participants (*M*_age_ = 19.6, *SD* = 1.55; 61 females) were included in the final sample for analyses. We followed Perugini et al. (2018) recommendations to compute the power analysis (see supplementary material for more details). This sample size enables to detect a medium (according to Cohen, 1988) or a large (according to Lovakov and Agadullina, 2021) effect size (*f* = 0.37) with a statistical power of 0.80 and α = 0.05.

### Materials

#### Activation of social norms

We manipulated the social-norm activation during the first normative-salience step of the IHP. We used the ingroup norm of antidiscrimination adapted from the work of Gabarrot et al. (2009). Participants were informed about the results of a supposed study carried out with a representative sample of students from their University. For the *descriptive norm* activation, two charts informed participants that most students did not discriminate against French people of foreign origin (FPFO). Specifically, results indicated that over 80% of them allocated resources between FPFO and French people of French origin in an egalitarian way. These resources concerned housing and education benefits. For the *injunctive norm* activation, two charts informed participants that most students did not agree with discrimination against FPFO. Specifically, results indicated that most students considered it illegitimate to favor French people of French origin over FPFO in terms of housing (89.26%) and education benefits (82.25%). For the *combined-norms* activation (i.e., both *descriptive* and *injunctive norms*), two graphs indicated that most students did not legitimize discrimination and did not themselves discriminate against FPFO. Specifically, one chart was used to show the same information as for the descriptive activation, and another one was used to show the same information as for the injunctive activation. Finally, to be sure that these supposed results were taken into account, participants were asked to answer a comprehension question (Smith and Louis, 2008).

#### Recall of past transgressions

Except participants of the control group, participants completed a questionnaire concerning their past behavior in five discrimination situations (i.e., criticizing, avoiding, keeping your distance from, staring at and being wary of a foreign person). They were asked to provide details about these situations, such as when it last happened, where they were, and who was concerned by the situation. This classically used questionnaire (Fried, 1998) facilitates participants’ transgression recall to make them conscious of their own counter-normative acts.

#### Dependent measures

We used measures of both active and passive discriminatory behaviors towards foreign people. We measured the participants’ normative behavioral intention (i.e., intention to engage in active promotion of antidiscrimination), which is the most classic mean for measuring the hypocrisy effect (Priolo et al., 2019). However, because of the limitations of this type of measure, which would not allow to show a reduction in participants’ discriminatory behaviors, we also used the exclusion of stigmatized targets in the Cyberball game (Pryor et al., 2013; Wesselmann et al., 2015) as a measure of passive discrimination against FPFO. The Cyberball game was chosen because it has several advantages. First, it offers a behavioral (here discriminatory) measure that can be used in the laboratory, whereas the use of behavioral measures in the induced-hypocrisy paradigm represents only a minority of studies (see Priolo et al., 2019). Second, unlike other measures of discriminatory behavior (e.g., allocation task, Anier et al., 2018 or organizational hierarchy task, Michinov et al., 2005), the Cyberball game allows to measure passive discriminations, related to the exclusion of a target. This makes the behavioral measure consistent with transgressions to be recalled by participants in the second step of the IHP (passive exclusion-type discriminations) and tests the effectiveness of the hypocrisy procedure on a form of discrimination that focuses researchers’ attention in terms of prevention. Third, the Cyberball game allows measuring deliberate but also less deliberate discriminatory behaviors (Pryor et al., 2013). Considering that we are activating social norms in our experimental procedure and that we know people are motivated to deliberately inhibit their discriminatory behaviors in order not to appear discriminatory (Devine et al., 2002), it seemed important to us to test the effect of our hypocrisy procedure on less deliberate behaviors.

##### Behavioral intention

Participants were asked to indicate how long they were willing to spend distributing flyers as members of an antidiscrimination association. Concretely, they were asked to indicate a number of half hours they wished to allow (between 0 and 8) as well as their first name, last name and their email-address to be contacted by the association. There was no set time period (e.g., 1 day, 1 week, 1 month) indicated to participants to achieve their volunteering. The higher the level of volunteering, the greater the hypocrisy effect.

##### Indicators of passive discriminatory behavior in the Cyberball game

**Figure 1:**
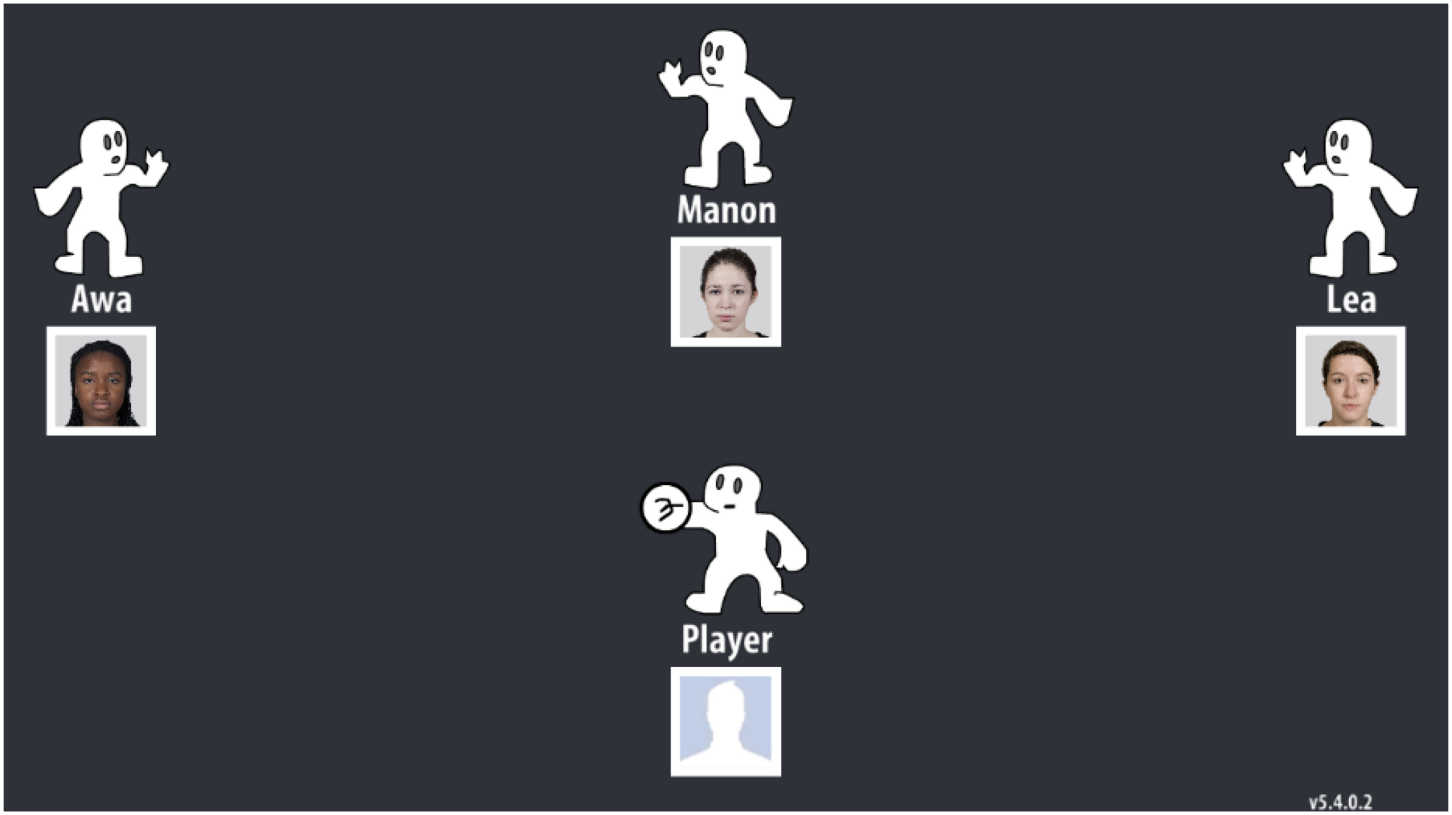
Illustration of the cyberball game for female participants. The bogus players’ images were taken from the Langner et al. (2010) radboud faces database. Reproduced with permission from Langner et al. (2010), available at: https://rafd.socsci.ru.nl/RaFD2/RaFD?p=main.

In the Cyberball game, participants were seated in front of a computer and were instructed to play a Cyberball game. This was described to participants as a mental visualization task where they were asked to imagine playing a real-life ball-tossing game with other participants. Participants were informed that they were playing with three other students from their University, who in real-life were three bogus players pre-programmed by the experimenter. On screen, each player was represented by an animated “Cyberboy” figure. Above or to the side of each player was a head-shot photo along with their first name. Using said photo and first name, one of the players was a black student (i.e., the target player), and the other two were white (see Figure 1 for female participants). To control the gender effect, all bogus players were men for male participants and women for female participants. Cyberball was programmed to carry on for 60 ball tosses and the three bogus players were programmed to equally included other participants. When participants received the ball, they elected to toss the ball to any of the players by clicking on their “Cyberboy” with the computer mouse.

The Cyberball game provided three indicators of discriminatory behavior (Pryor et al., 2013). The first one was the Cumulative Number of Tosses (CNT) to the black player across the entire game. The lower the CNT was, the greater the discrimination rate was. The second one was the Number of Tosses to white players Before Including the black player in the game (NTBI). The greater the NTBI was, the greater the discrimination rate was. These first two indicators reflected participants’ deliberate and conscious choices. The third indicator was the Hesitation Given Inclusion (HGI) and represented the average latency across trials in throwing the ball to each player. The difference between the HGI for the black player and the mean HGI for the white players was used to measure less deliberate and less conscious behaviors. The higher the HGI was, the greater the discrimination rate was.

### Design and procedure

Participants were approached individually on the campus. After agreeing to participate, students were randomly assigned to one of four conditions. In the control condition, the dependent measures were directly proposed to the participants. In the three other hypocrisy conditions, namely descriptive norm-related hypocrisy, injunctive norm-related hypocrisy, and combined-norm hypocrisy, we activated the descriptive or injunctive normative beliefs or a combination of both. Then, participants completed the transgressions-salience step and, finally, the dependent variables. We debriefed all the participants in order to detect any suspicion about Cyberball (i.e., a realistic game with real persons). All the participants were convinced of this, except for one, who was removed from the analyses as previously indicated. Finally, participants were thanked for their participation.

### Hypotheses and data analysis

First, we expected that participants’ time given to association (H1) would be greater in the combined-norm hypocrisy condition compared to descriptive norm-related hypocrisy and injunctive norm-related hypocrisy conditions, which would be greater than in the control condition. Second, we expected in the Cyberball game the number of throws to the target (CNT) to increase (H2), and the number of throws before target inclusion (NTBI) and the difference in time to include the target compared to the other two players (HGI) to decrease (H3 and H4) as follows: control condition, then descriptive norm-related hypocrisy and injunctive norm-related hypocrisy conditions, and finally, the combined-norm hypocrisy condition.

We analyzed data as described in the following two steps. In the first step, to test the effects of the norms in isolation, we ran 2 × 2 ANOVAs with the following design: Injunctive norms (Present vs. Absent) × Descriptive norms (Present vs. Absent). In the second step, we tested our specific hypotheses. We used the contrast method as recommended by many authors (Brauer and McClelland, 2005; Abdi and Williams, 2010; Judd et al., 2017), with the first contrast (i.e., interest contrast) testing our hypothesis (control = −1; descriptive norm-related hypocrisy = 0; injunctive norm-related hypocrisy = 0; combined-norm hypocrisy = 1) and the two others being residuals (C2: control = 0; descriptive norm-related hypocrisy = −1; injunctive norm-related hypocrisy = 1; combined-norms hypocrisy = 0; and C3: control = 1 descriptive norm-related hypocrisy = −1; injunctive norm-related hypocrisy = −1; combined-norms hypocrisy = 1). To conclude that the data were consistent with our hypotheses, three conditions had to be satisfied (Brauer and McClelland, 2005). The contrast of interest had to explain a significant part of the variance of the dependent variables while the two residuals had to be non-significant (i.e., *p* > 0.05).

## Results

As the number of transgressions recalled impacts the hypocrisy effect (Fointiat et al., 2008; Stone and Fernandez, 2011; Sénémeaud et al., 2014), we first verified that it did not vary across the three hypocrisy conditions (see supplementary material for statistical results).

Descriptive data are presented in Table 1 and statistical results of ANOVAs and linear regressions are displayed in Table 2.

**Table 1:** Means, standard deviations, and ratio for study variables across conditions.

Conditions	Measures	Volunteering		CNT	NTBI		HGI	
		*M*	*SD*	*Ratio*	*M*	*SD*	*M*	*SD*
Control		10.5	22.3	0.298	1.2	0.52	0.298	1.7
Descriptive norm-related hypocrisy		30	41.4	0.292	1.1	0.55	0.292	1.01
Injunctive norm-related hypocrisy		25.5	38.1	0.313	1.05	0.61	0.313	1.03
Combined-norm hypocrisy		45	42.9	0.324	0.8	0.77	0.324	0.918

*N* = 80. For volunteering, number of tosses before inclusion (NTBI) and hesitation given inclusion (HGI), data show the mean (standard deviation). For cumulative number of tosses (CNT), data show the ratio (number of tosses to the black player/number of tosses made in the game).

**Table 2:** Omnibus effects and planned comparisons for 2 × 2 ANOVAs on the four dependent variables.

	**Volunteering**		**CNT**		**NTBI**		**HGI**	
		*F* (η^2^)	Estimate (*SE*)	*F* (η^2^)	Estimate (*SE*)	*F* (η^2^)	Estimate (*SE*)	*F* (η^2^)	Estimate (*SE*)
*Omnibus effects*								
Injunctive-norm	3.27 (0.039)		4.12* (0.051)		2.64 (0.33)		3.88 (0.045)	
Descriptive-norm	5.53* (0.065)		0.042 (0.00)		1.60 (0.02)		5.82** (0.067)	
INxDN interaction	0.0 (0.00)		0.56 (0.007)		0.29 (0.004)		1.09 (0.013)	
*Planned comparisons*								
Contrast of interest		0.320** (0.391)		0.026 (0.016)		−0.400* (0.196)		−1.18** (0.381)
Residual contrast 1		−0.042 (0.391)		0.021 (0.016)		−0.050 (0.196)		0.119 (0.381)
Residual contrast 2		−0.001 (0.553)		0.017 (0.023)		−0.150 (0.277)		−0.563 (0.539)

Statistically significant at * *p* < 0.05; ** *p* < 0.01; *** *p* < 0.001.

### Omnibus effects and planned comparisons on the amount of volunteering

**Figure 2:**
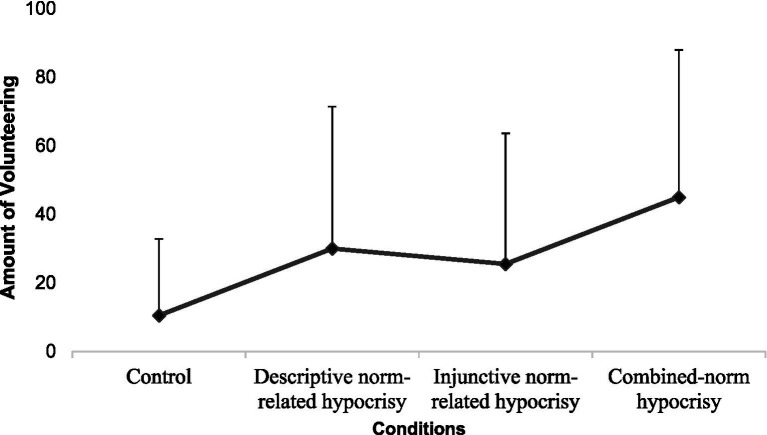
Participants’ amount of volunteering. Volunteering of participants is shown for the four experimental conditions (*N* = 80). Volunteering was measured by asking participants how much time they were willing to give to a discrimination prevention association. Volunteering is in minutes and error bars show standard deviations.

First, the omnibus model indicated a significant main effect of Descriptive-norm variable [*F*(1,76) = 5.53, *p* = 0.021, η^2^ = 0.065], and no significant main effect of Injunctive-norm variable [*F*(1,76) = 3.27, *p* = 0.074, η^2^ = 0.039] as well as no interaction effect [*F*(1,76) = 0.00, *p* = 0.1, η^2^ = 0.00]. Second, results of our planned comparisons indicated that the contrast of interest is significant, *t* = 2.94, *p* = 0.004, *Estimate* = 1.150, 95% CI [0.372, 1.928], and the two residual contrasts were not significant (see Table 2). Thus, results were consistent with our hypothesis (H1) showing a positive trend in participants’ amount of volunteering from the control condition to the conditions of descriptive norm-related hypocrisy and injunctive norm-related hypocrisy and finally, the combined-norm hypocrisy condition (see Figure 2).

### Omnibus effects and planned comparisons on CNT

First, the omnibus model indicated a significant main effect of Injunctive-norm variable [*F*(1,76) = 4.12, *p* = 0.046, η^2^ = 0.051], and no significant main effect of Descriptive-norm variable [*F*(1,76) = 0.042, *p* = 0.84, η^2^ = 0.00] as well as no interaction effect [*F*(1,76) = 0.56, *p* = 0.46, η^2^ = 0.007]. Second, results of our planned comparisons indicated neither significant effect for the contrast of interest, *t* = 1.58, *p* = 0.12, *Estimate* = 0.026, 95% CI [−0.007, 0.058], nor for the both residual contrasts (see Table 2). These results do not support our hypothesis (H2) as we do not observe a significant positive trend in participants’ cumulative number of tosses for the black target from the control condition to the conditions of descriptive norm-related hypocrisy and injunctive norm-related hypocrisy and finally, the combined-norm hypocrisy condition.

### Omnibus effects and planned comparisons on NTBI

First, the omnibus model indicated no significant main effects of Injunctive-norm [*F*(1,76) = 2.64, *p* = 0.11, η^2^ = 0.033] and Descriptive-norm variable [*F*(1,76) = 1.60, *p* = 0.21, η^2^ = 0.020], and no interaction effect [*F*(1,76) = 0.29, *p* = 0.59, η^2^ = 0.004]. Second, results of our planned comparisons indicated a significant effect for the contrast of interest, *t* = −2.04, *p* = 0.045, *Estimate* = −0.40, 95% CI [−0.79, −0.010], but no significant effects for the both residual contrasts (see Table 2). Thus, results were consistent with our hypothesis (H3) showing a negative trend in participants’ number of tosses before including the black target from the control condition to the descriptive norm-related hypocrisy and injunctive norm-related hypocrisy conditions and finally, the combined-norm hypocrisy condition.

### Omnibus effects and planned comparisons on HGI

**Figure 3:**
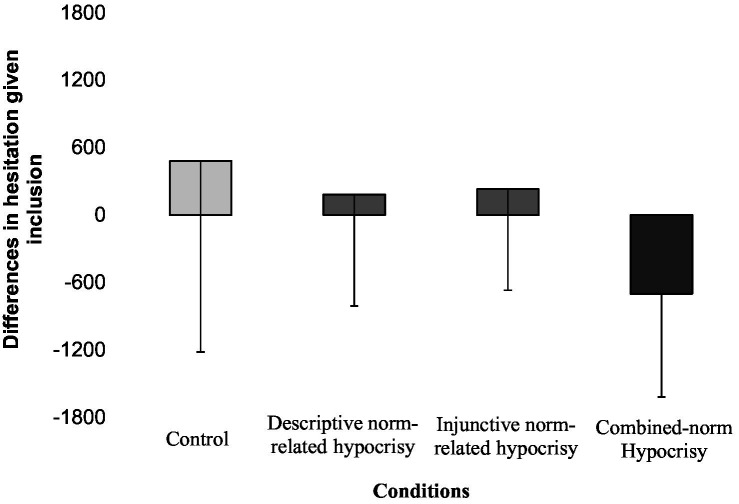
Differences in participants’ hesitation given inclusion. Differences in participants’ hesitation given inclusion (HGI) between the black player and the white players are shown for the four experimental conditions (*N* = 80). Differences in hesitation given inclusion were calculated by subtracting the average HGI scores of the white players from the HGI score of the black player. Scores are in milliseconds and error bars show standard deviations.

First, the omnibus model indicated a significant main effect of Descriptive-norm variable [*F*(1,76) = 5.82, *p* = 0.018, η^2^ = 0.067], and no significant main effect of Injunctive-norm variable [*F*(1,76) = 3.88, *p* = 0.052, η^2^ = 0.045] as well as no interaction effect [*F*(1,76) = 1.09, *p* = 0.30, η^2^ = 0.013]. Second, results of our planned comparisons indicated a significant effect for the contrast of interest, *t* = −3.10, *p* = 0.003, *Estimate* = −1.18, 95% CI [−1.94, −0.42], but no significant effects for the both residual contrasts (see Table 2). Thus, results were consistent with our hypothesis (H4) showing a negative trend in participants’ hesitation given inclusion the black target from the control condition to the descriptive norm-related hypocrisy and injunctive norm-related hypocrisy conditions and finally, the combined-norm hypocrisy condition (see Figure 3).

## Discussion

This study had two objectives. First, we tested, within the IHP framework, the effect of perceived deviance from the antidiscrimination norm on reduction of discriminatory behaviors. Second, we examined whether IHP effects can be reinforced by the activation of the deviance from both injunctive and descriptive norms. Overall, results first show that the hypocrisy effect on behavioral antidiscrimination intention and passive discriminatory behaviors is reproduced. Indeed, recalling the antidiscrimination social norm (whether it is injunctive, descriptive or both) and past behaviors deviating from the norm, leads the participants to actively promote antidiscrimination and to reduce their discriminatory behaviors. Second, results showed that the combined use of descriptive and injunctive norms in the IHP enhanced this hypocrisy effect, leading participants to further reduce their discriminatory behaviors. These results will be discussed with regard to two major implications. First, they provide a new and effective means to produce behavioral changes in the field of discrimination. Second, they contribute to further investigating the explanatory processes underlying the hypocrisy effect.

First, while IHP had demonstrated its effectiveness in many fields (see Priolo et al., 2019), it has only been tested once in the field of discrimination (Son Hing et al., 2002). Indeed, Son Hing et al. (2002) had shown that IHP could reduce budget restrictions among Asian students. Our study completes their findings, 20 years later, by showing that hypocrisy can also reduce interpersonal discrimination against French people from a foreign origin. According to us, this IHP applicability to the prevention of interpersonal discrimination is interesting in two ways. On the one hand, our results showed that the IHP impacts not only deliberate (i.e., behavioral intentions and NTBI indicator) but also less controllable discriminatory behaviors (i.e., HGI indicator in Cyberball, Pryor et al., 2013). These results are particularly interesting and innovative in the discrimination field, because more subtle, less deliberate discrimination remains an issue (Molero et al., 2013). They are also innovative when it comes to IHP field. Indeed, the hypocrisy effect is classically observed on conscious behaviors (Liégeois et al., 2017). Apart from a few studies showing behavioral changes when people were unaware that their behaviors were measured (Dickerson et al., 1992), our study is the first to demonstrate that the IHP can impact unintentional and uncontrollable behaviors. The Associative-Propositional Evaluation model (Gawronski and Bodenhausen, 2006) could shed some light on this result. One might think that dissonance would not be totally reduced by the conscious way, needing to reduce the residual part of dissonance by another means (a less conscious routeway). In the IHP, a single dissonance reduction route is sometimes not sufficient (Fointiat et al., 2013). In any case, additional data are needed to specifically address this issue of less deliberate and unconscious behavioral change following the IHP. On the other hand, we believe that the IHP, because it fosters awareness of one’s deviant behaviors, may be a necessary and therefore crucial preliminary step to regulate discriminatory behaviors. According to the self-regulation model of prejudice (Monteith et al., 1993, 2016), individuals are likely to perceive that their behaviors deviate from antidiscrimination norms. This perception would lead them to be willing to act “better” in the future (i.e., not to discriminate). They indeed become sensitive to environmental cues that may trigger these discriminatory behaviors, and thus can suppress and replace them with adapted and prejudice-free behaviors. However, two conditions appear to be important for this self-regulation process to take place: (i) people must be motivated to regulate and reduce their discriminatory behaviors (Devine et al., 2002) and (ii) people need to identify that their behaviors are discriminatory and counter-normative - which is not obvious for passive and subtle discriminations (Kite and Whitley, 2016). Although this first condition was not experimentally manipulated in our study, our proposed idea is that the IHP would fulfill both of these conditions. On the one hand, it fosters people’s awareness of their indirect and subtle counter-normative behaviors by asking them to recall it. On the other hand, it helps motivate people to act in line with the norm. Thus, the hypocrisy paradigm seems to us a promising tool in the field of discrimination prevention and particularly in terms of reducing passive discrimination, which is currently the main issue. It should be further developed in order to increase people’s motivation to effectively regulate their own discriminatory behaviors.

Second, our focus on the role of social norms as a reinforcer of behavioral change in the induced-hypocrisy paradigm addresses a gap in IHP literature. A great deal of research has investigated the role of the transgressions-salience step at the expense of the normative-salience step (see Stone and Fernandez, 2008 for a review). By showing the reinforcing role of joint activation of social norms in the normative-salience step on the hypocrisy effect, we first contribute to identifying the optimal conditions for applying the paradigm. More critically, we further our understanding of the processes underlying the hypocrisy effect. Indeed, the explanation in terms of self-consistency (Aronson, 1999) has been prevalent since the paradigm’s genesis and has rarely been challenged, except by the deviation-from-norm approach (Liégeois et al., 2017). A more integrative explanation has also been developed, based on the Self-Standard Model of dissonance (SSM, Stone and Cooper, 2001; Stone and Fernandez, 2008). According to the latter, induced hypocrisy could be explained either by a first pathway, that of the threat to self (i.e., a self-consistency effect) or by a second pathway, that of the deviation from the social norm. However, this second pathway clearly lacks experimental support. We suggest that our study may be one of the first experimental evidence of its existence. More specifically, according to Thøgersen (2006) taxonomy of norms, descriptive norms are integrated into the self in a lesser extent than injunctive norms. Therefore, under a self-consistency view (the first pathway in Stone and Cooper’s SSM), the injunctive norm should have caused a greater *hypocrisy effect* than the descriptive norm because the more the self is threatened, the higher the hypocrisy effect. In this perspective too, the combined use of two norms should not have caused a greater effect than the injunctive. Yet, we observed exactly the opposite. Therefore, our results are consistent with the second pathway of SSM and the deviation-from-norm approach (Liégeois et al., 2017) which suggests that the hypocrisy effect could not only be due to self-threat but also to the awareness of norm deviation. Our further research will attempt to provide additional experimental evidence for this social norm deviation pathway, such as testing whether weakening social norms in the normative step reduces the hypocrisy effect.

### Limitations

First, our sample was composed only of participants who agreed to take part in an interpersonal relations study. This in itself may constitute a bias of self-selection. Second, we do not reach sufficient statistical power for some study measures. As some researchers (e.g., Christley, 2010) suggest that underpowered studies increase the likelihood of making type I error, future research should attempt to replicate this additive effect of combining two norms rather than one in the hypocrisy procedure. Third, significant results consistent with our hypotheses were obtained on three of four indicators, the effect of our experimental procedure was not significant on the Cyberball CNT indicator. The fact the CNT is a deliberate behavioral measure (i.e., to give or not to give the ball to the target) could explain it. While we know that people are motivated to inhibit the expression of their prejudices and discriminatory behaviors (e.g., Devine et al., 2002), participants have completed the Cyberball game after responding to the behavioral intention measure (volunteering to a discrimination prevention association) which may have made salient the social norm against discrimination. All of this may have (i) weakened the effect of our hypocrisy procedure on this deliberate measure and especially (ii) led participants in the control condition to not deliberately discriminate against the target. Our results seem to support this assumption since the participants in the control condition sent the ball on average 29.8% of the time to the target (33% being a fair distribution). A ceiling effect could have been observed on this measure, not allowing to test the hypocrisy effect in optimal conditions. Therefore, future research could attempt to replicate the hypocrisy effect on discriminatory behavior by using the Cyberball game directly after the hypocrisy procedure. Fourth, our study is about the influence of group norms on behaviors and we know that the individual’s group identity can moderate it. It would be interesting to assess the participants’ level of identification with the group to better understand our results.

### Conclusion

Societal changes in terms of antidiscrimination norms have led people to inhibit the expression of their prejudices and discriminatory behaviors. People could thus be reluctant to recall them. This is probably the reason why the hypocrisy paradigm has rarely been applied to the field of discrimination prevention, since it is largely applied to transgressive behaviors that are easy to remember and recall. In this case, reinforcing the first IHP step of normative salience may be necessary to consider for inducing people’s sense of hypocrisy. Our study suggests that the IHP may also be an effective solution for preventing discrimination if, without the realization of an optimal transgression-recall step, deviance from the norm is increased by reinforcing the anti-normative content of discrimination during the normative step.

## Data availability statement

The datasets presented in this study can be found in online repositories. The names of the repository/repositories and accession number(s) can be found at: https://osf.io/ryg8t/?view_only=90e6af6fa1994e0f940e85fc09390779. Preregistration details are available at https://doi.org/10.17605/OSF.IO/HGB6W.

## Ethics statement

Ethical review and approval was not required for the study on human participants in accordance with the local legislation and institutional requirements. The patients/participants provided their written informed consent to participate in this study.

## Author contributions

All authors have read and approved the manuscript for submission to Frontiers in Psychology; have made a substantial contribution to the conception, design, data collection, analysis and/or interpretation as well as a contribution to the drafting and intellectual content of the article; and acknowledge that they have exercised due care in ensuring the integrity of the work. The authorship reflects the contribution of each author except for Cécile Sénémeaud who coordinates this work.

## Funding

The work reported was supported by RIN Doctorant of Normandie Région, France. Open access funding provided by University of Lausanne.

## Conflict of interest

The authors declare that the research was conducted in the absence of any commercial or financial relationships that could be construed as a potential conflict of interest. The reviewer LR declared a shared research group with the author(s) to the handling Editor.

## Publisher’s note

All claims expressed in this article are solely those of the authors and do not necessarily represent those of their affiliated organizations, or those of the publisher, the editors and the reviewers. Any product that may be evaluated in this article, or claim that may be made by its manufacturer, is not guaranteed or endorsed by the publisher.
