# Supplementary material for: When combining injunctive and descriptive norms strengthens the hypocrisy effect: A test in the field of discrimination
Source: Front Psychol. 2022 Nov 29;13:989599. doi: 10.3389/fpsyg.2022.989599 (PMC9746236; doi:10.3389/fpsyg.2022.989599)
Supplement: Supplementary file 1 [file Data_Sheet_1.PDF]

Supplementary Material

Article: "When Combining Injunctive and Descriptive Norms Strengthens the Hypocrisy Effect:

A Test in the Field of Discrimination"

1 Statistical Power

A priori power analysis for a continuous dependent variable was performed. As we planned to use the contrast method, Perugini et al.' (2018) recommendations to compute the power analysis for contrasts were followed. One possible solution was to specify the expected pattern of means for the interest contrast and to use the standardized means to compute the expected effect size. Our computation of effect sizes was based on the meta-analysis of Priolo et al. (2019). In this paper, the effect size of induced hypocrisy is estimated to be $r = .35$. We converted this effect size into Cohen's d and it was equal to $d = 0.75$. Considering that meta-analyses can report inflated effect sizes, we decided to use $d = 0.65$ as the effect size for induced hypocrisy.

Concretely, we fixed the means to *injunctive hypocrisy* and *descriptive hypocrisy* conditions to 0. The pooled standard deviation was equal to 1. We set the *control* group's mean at -0.65 standard deviation from the *injunctive* and *descriptive hypocrisy* conditions' means, and the mean of the combined norms hypocrisy at + 0.65 standard deviations from the means of the other hypocrisy conditions. The computed expected f was equal to 0.42. Thus, the sensitivity analysis with G*power revealed that our sample size of 80 participants enabled a sufficient statistical power of 0.88 (with $f = .42$ and $\alpha = .05$) to be achieved[1]. This analysis revealed that we could detect a medium (according to Cohen, 1988) or a large (according to Lovakov & Agadullina, 2021) effect size ($f = .37$) with a statistical power fixed at 80%.

---

[1] It should be noted that this study was initially pre-registered with 140 participants to analyze a binary variable (i.e., the target request acceptance). Due to the COVID-19 pandemic, we had to halt the procurement process study at N = 80.

2 Analysis of Variance (ANOVA) on the Number of Transgressions Recalled

We conducted an ANOVA on the number of transgressions recalled with the *hypocrisy* conditions as the between-participants factor. The analysis revealed that the number of transgressions did not differ across the three *hypocrisy* conditions, $F(2, 57) = 0.74$, $p = .48$, $\eta^2_p = .025$ ($M_{\text{descriptive hypocrisy}} = 2.70$, $SD = 1.42$; $M_{\text{injunctive hypocrisy}} = 2.25$, $SD = 1.21$; $M_{\text{combined-norms hypocrisy}} = 2.35$, $SD = 1.04$).
